# A112 OUTCOMES OF PATIENTS AT INCREASED RISK FOR GASTRIC NEOPLASIA: A RETROSPECTIVE COHORT STUDY

**DOI:** 10.1093/jcag/gwae059.112

**Published:** 2025-02-10

**Authors:** D Koerber, C Roda, S Pang, P Tavakoli, M Ten-Pow, A Walia, E Lam, D Motomura, W Xiong, N Shahidi

**Affiliations:** The University of British Columbia Faculty of Medicine, Vancouver, BC, Canada; The University of British Columbia Faculty of Medicine, Vancouver, BC, Canada; StPaul’s Hospital Division of Gastroenterology, Vancouver, BC, Canada; StPaul’s Hospital Division of Gastroenterology, Vancouver, BC, Canada; StPaul’s Hospital Division of Gastroenterology, Vancouver, BC, Canada; StPaul’s Hospital Division of Gastroenterology, Vancouver, BC, Canada; StPaul’s Hospital Division of Gastroenterology, Vancouver, BC, Canada; StPaul’s Hospital Division of Gastroenterology, Vancouver, BC, Canada; University of British Columbia Department of Pathology and Laboratory Medicine, Vancouver, BC, Canada; StPaul’s Hospital Division of Gastroenterology, Vancouver, BC, Canada

## Abstract

**Background:**

Gastric cancer is the third most common cause of cancer-related deaths, with 784,000 deaths annually and a 26% five-year overall survival. In contrast, the five-year survival in Japan exceeds 70%, which is in part attributed to increased awareness and surveillance for patients and increased risk of gastric cancer.

**Aims:**

We aimed to evaluate outcomes of patients at increased risk of gastric cancer in a North American tertiary referral center.

**Methods:**

Between 01/2012 to 07/2024, consecutive patients with gastric atrophy, intestinal metaplasia, and low-grade or high-grade gastric dysplasia were identified using a validated histopathology registry at St. Paul’s Hospital (Vancouver, BC, Canada). Full chart review was then completed, including demographic and outcome variables. Continuous variables were summarised using median (IQR) and categorical variables were summarised as frequencies (%).

**Results:**

Preliminary analysis from 01/2012 to 5/2014 identified 127 patients: 8 (6%) gastric atrophy, 114 (90%) gastric intestinal metaplasia, 5 (4%) low- or high-grade dysplasia. Median patient age was 65 (56-75) years, with 61 (48%) being female. Concomitant Helicobacter pylori was identified in 34 (27%) with 12 (9%) receiving eradication therapy and confirmation of eradication. Follow-up data was available for a median of 5.7 (0.4-10.3) years. Endoscopic surveillance was performed in 38 (30%) patients. Gastric cancer was not identified during surveillance, with 1 (1%) patient developing dysplasia.

**Conclusions:**

Patients at increased risk of gastric cancer represent a significant clinical burden within tertiary gastroenterology centers. Gastric cancer risk commonly goes unrecognized leading to low rates of endoscopic surveillance. Programmatic management may lead to optimized outcomes and resource utilization.

**Funding Agencies:**

**None**

